# Treatment Strategies and Survival of Older Breast Cancer Patients – An International Comparison between the Netherlands and Ireland

**DOI:** 10.1371/journal.pone.0118074

**Published:** 2015-02-03

**Authors:** Mandy Kiderlen, Paul M. Walsh, Esther Bastiaannet, Maria B. Kelly, Riccardo A. Audisio, Petra G. Boelens, Chris Brown, Olaf M. Dekkers, Anton J. M. de Craen, Cornelis J. H. van de Velde, Gerrit-Jan Liefers

**Affiliations:** 1 Dept. of Surgery, Leiden University Medical Center, Leiden, The Netherlands; 2 Dept. of Gerontology & Geriatrics, Leiden University Medical Center, Leiden, The Netherlands; 3 National Cancer Registry of Ireland, Cork, Ireland; 4 Department of Surgery, St Helens Teaching Hospital, St Helens, United Kingdom; 5 University of Liverpool, Liverpool, United Kingdom; 6 Dept. of Epidemiology, Leiden University Medical Center, Leiden, The Netherlands; Sudbury Regional Hospital, CANADA

## Abstract

**Objectives:**

Forty percent of breast cancers occur among older patients. Unfortunately, there is a lack of evidence for treatment guidelines for older breast cancer patients. The aim of this study is to compare treatment strategy and relative survival for operable breast cancer in the elderly between The Netherlands and Ireland.

**Material and Methods:**

From the Dutch and Irish national cancer registries, women aged ≥65 years with non-metastatic breast cancer were included (2001-2009). Proportions of patients receiving guideline-adherent locoregional treatment, endocrine therapy, and chemotherapy were calculated and compared between the countries by stage. Secondly, 5-year relative survival was calculated by stage and compared between countries.

**Results:**

Overall, 41,055 patients from The Netherlands and 5,826 patients from Ireland were included. Overall, more patients received guideline-adherent locoregional treatment in The Netherlands, overall (80% vs. 68%, adjusted p<0.001), stage I (83% vs. 65%, p<0.001), stage II (80% vs. 74%, p<0.001) and stage III (74% vs. 57%, P<0.001) disease. On the other hand, more systemic treatment was provided in Ireland, where endocrine therapy was prescribed to 92% of hormone receptor-positive patients, compared to 59% in The Netherlands. In The Netherlands, only 6% received chemotherapy, as compared 24% in Ireland. But relative survival was poorer in Ireland (5 years relative survival 89% vs. 83%), especially in stage II (87% vs. 85%) and stage III (61% vs. 58%) patients.

**Conclusion:**

Treatment for older breast cancer patients differed significantly on all treatment modalities between The Netherlands and Ireland. More locoregional treatment was provided in The Netherlands, and more systemic therapy was provided in Ireland. Relative survival for Irish patients was worse than for their Dutch counterparts. This finding should be a strong recommendation to study breast cancer treatment and survival internationally, with the ultimate goal to equalize the survival rates for breast cancer patients across Europe.

## Introduction

Currently, about 40 per cent of all new breast cancer cases in developed countries occur among women aged 65 and older [1]. Life expectancy is increasing, diagnostic tools become more sensitive and screening programs are more widely used and expanded. Consequently, the proportion of elderly breast cancer patients is expected to increase in the near future [2].

Proper treatment for older breast cancer patients is difficult to define. Older women are frequently excluded from clinical treatment trials because of their age, comorbidity or logistical barriers [3]. Moreover, the elderly who are included in trials are probably not representative for the general older population [4]. Consequently, an evidence-based treatment strategy for older women with breast cancer is lacking. The only guidance for clinicians is from treatment guidelines which have been validated in younger and healthier women [5]. Extrapolation from trials might not be valid since breast cancer biology differs in some respects in older patients, treatment tolerance varies, and there are substantial competing risks of mortality [2,6]. Consequently, clinicians have to decide what is best for their patient: treatment according to the guidelines, or patient-tailored deviation from the guidelines.

In the last decade it has become more accepted to use observational data, preferably population-based, to assess treatment effects in older cancer patients [7]. However, no strong conclusions can be drawn from these studies as bias due to confounding by indication is likely to be present, since specific (unknown) patient and tumor-related factors influence receipt of particular treatments.[8]

A recent observational study comparing locoregional treatment between six European countries and the US found that treatment strategy in The Netherlands and Ireland differed considerably on various items among older women with early stage breast cancer, indicating that older patients with early stage breast cancer in Ireland seemed to be slightly undertreated, compared with The Netherlands. However, relative survival was not demonstrably different [9].

The aim of the present study is to compare treatment strategy and relative survival for operable (non-metastatic) breast cancer in the elderly between The Netherlands and Ireland in more detail.

## Materials and Methods

### Data

From the Netherlands and Irish cancer registry, all female patients aged 65 years and older diagnosed between 2001 and 2009 with invasive, non-metastasized breast cancer were selected. Patients with a diagnosis of breast cancer on death certificate or at autopsy only, and other patients with a survival time of zero days, were excluded. If a patient had a second primary tumor during follow-up, only the first primary breast tumor was considered for analyses.

Tumor stage was defined by TNM stage [10], with clinical T and N used when pathological information was lacking. Patients with missing T category were excluded. When nodal and distant metastatic status were unspecified (NX and MX), status was assumed to be N0 and M0, respectively. Stage data were originally coded using 6th-edition TNM [10] rules in the Netherlands and 5th-edition TNM [11] rules in Ireland. Micrometastases (≤0.2 cm) in regional nodes, classified as N1a in 5th-edition TNM were recoded to N0 for 21 Irish cases to conform to 6th-edition TNM rules. For surgical treatment, only the most extensive surgery registered was used for analysis. Axillary surgery was coded as yes or no.

Primary outcome was treatment strategy by stage. Treatments of interest were type of surgery (none, BCS or mastectomy), radiotherapy (RT; yes or no), axillary surgery (yes or no), locoregional guideline adherence (details below), endocrine therapy (yes or no) and chemotherapy (yes or no). Secondary outcome measure was 5-year relative survival in each country.

In both the Dutch and Irish breast cancer guidelines, primary surgical treatment with mastectomy or BCS followed by radiotherapy (RT) is recommended for non-metastasized breast cancer. In addition, it is recommended to assess axillary nodal status by performing a sentinel node procedure or axillary lymph node dissection [12–14] (S1 Appendix). Therefore, locoregional treatment was considered guideline-adherent when a patient had BCS and RT or mastectomy with or without RT, in all cases followed by any axillary surgical procedure. In addition the receipt of systemic therapy (adjuvant endocrine therapy and chemotherapy) was analyzed.

Routine cancer registry data on endocrine therapy in Ireland were known to be incomplete (National Cancer Registry of Ireland, unpublished data), because of difficulties associated with outpatient prescription of the drugs involved. Endocrine therapy data for Irish patients were therefore supplemented by linkage to a national database of drug prescription, which covers publicly funded ‘medical card’ patients including most patients aged 65 years and over. Additional endocrine therapy was identified by this linkage for 21% of patients. Linkage was not possible for about 15% of Irish patients, and for this group, ‘missing’ endocrine therapy was imputed (4% of all patients). The imputation assumed that the proportion of ‘linked’ patients receiving endocrine therapy by stage (I, II and III), hormone receptor status (any positive vs. none positive) and broad age-group (65–74 and 75+) also applied to unlinked patients, and these ‘extra’ treatments were assigned randomly within each stage-by-age group.

Data from both the Netherlands Cancer Registry and the National Cancer Registry of Ireland are fully anonymized prior to being made available to researchers, so data cannot be traced back to the individual patient. Therefore, no informed consent was required from the included patients and there was no need for approval of an ethical committee.

Mortality follow-up was available to December 31^st^ 2011 by linkage of cancer registry with national mortality data.

### Statistical analyses

Analyses were performed in IBM SPSS Statistics 20 and Stata SE 12. Treatment strategies were analyzed grouped by tumor stage (I to III). Differences in treatment between countries were tested by a Poisson regression model, adjusted for age (continuous), histological subtype, tumor grade, ER and PR status.

Relative survival was calculated by the Ederer II method [15] as the ratio of the survival observed among the cancer patients to the expected survival based on the corresponding general population (by age, sex, and year of diagnosis), using the ‘strs’ command in Stata. National life tables for each country were used to estimate expected survival. Results were presented as percentage relative survival after five years, and Relative Excess Risks (RER) derived from relative survival modeling, with The Netherlands as reference category [16].

## Results

Overall, 41,055 patients from The Netherlands and 5,826 patients from Ireland were included. Patient and tumor characteristics are shown in Table 1. Median age for patients in The Netherlands was 74 years (range 65–102), and in Ireland 74.2 (range 65–99). Fewer early stage tumors, and more with advanced stage were observed in Ireland (*P*<0.001). Recorded grade distribution differed significantly, with a higher proportion of higher grades in Ireland than in The Netherlands (*P*<0.001).

**Table 1:** Patient en tumor characteristics.

		Country				
		The Netherlands		Ireland		
		(N = 41055)		(N = 5826)		
		N	%	N	%	P
Age (years)	65–74	22,036	53.7	3,126	53.7	0.989
	75 or older	19,019	46.3	2,700	46.3	
Year of diagnosis	2001	4,432	10.8	584	10.0	0.333
	2002	4,256	10.4	582	10.0	
	2003	4,339	10.6	601	10.3	
	2004	4,439	10.8	624	10.7	
	2005	4,425	10.8	614	10.5	
	2006	4,519	11.0	664	11.4	
	2007	4,870	11.9	695	11.9	
	2008	4,914	12.0	718	12.3	
	2009	4,861	11.8	744	12.8	
Stage	I	17,790	43.3	1,658	28.5	<0.001
	II	18,023	43.9	3,140	53.9	
	III	5,242	12.8	1,028	17.6	
Grade	1	8,137	19.8	542	9.3	<0.001
	2	16,314	39.7	2,803	48.1	
	3	9,018	22.0	1,720	29.5	
	missing	7,586	18.5	761	13.1	
Morphology	ductal	28,463	69.3	3,861	66.3	<0.001
	lobular	5,488	13.4	789	13.5	
	mixed/other	7,104	17.3	1,176	20.2	
ER	negative	3,209	7.8	930	16.0	<0.001^a^
	positive	19,785	48.2	4,074	69.9	
	missing	18,061	44.0	822	14.1	
PR	negative	7,350	17.9	1,545	26.5	<0.001 ^a^
	positive	14,740	35.9	2,694	46.2	
	missing	18,965	46.2	1,587	27.2	

^a^ Excluding missing values.

Hormone receptor status showed smaller differences, with slightly smaller proportions of estrogen and progesterone receptor positive tumors among Irish patients (81% and 64%, respectively, excluding missing or unknown values) compared with those from the Netherlands (86% and 67%) (*P* <0.001). The proportion of missing values was much lower in Ireland, mainly because Dutch data were not complete for the years 2001–2005 rather than to differences in proportions of patients tested.

### Locoregional treatment

**Fig 1:**
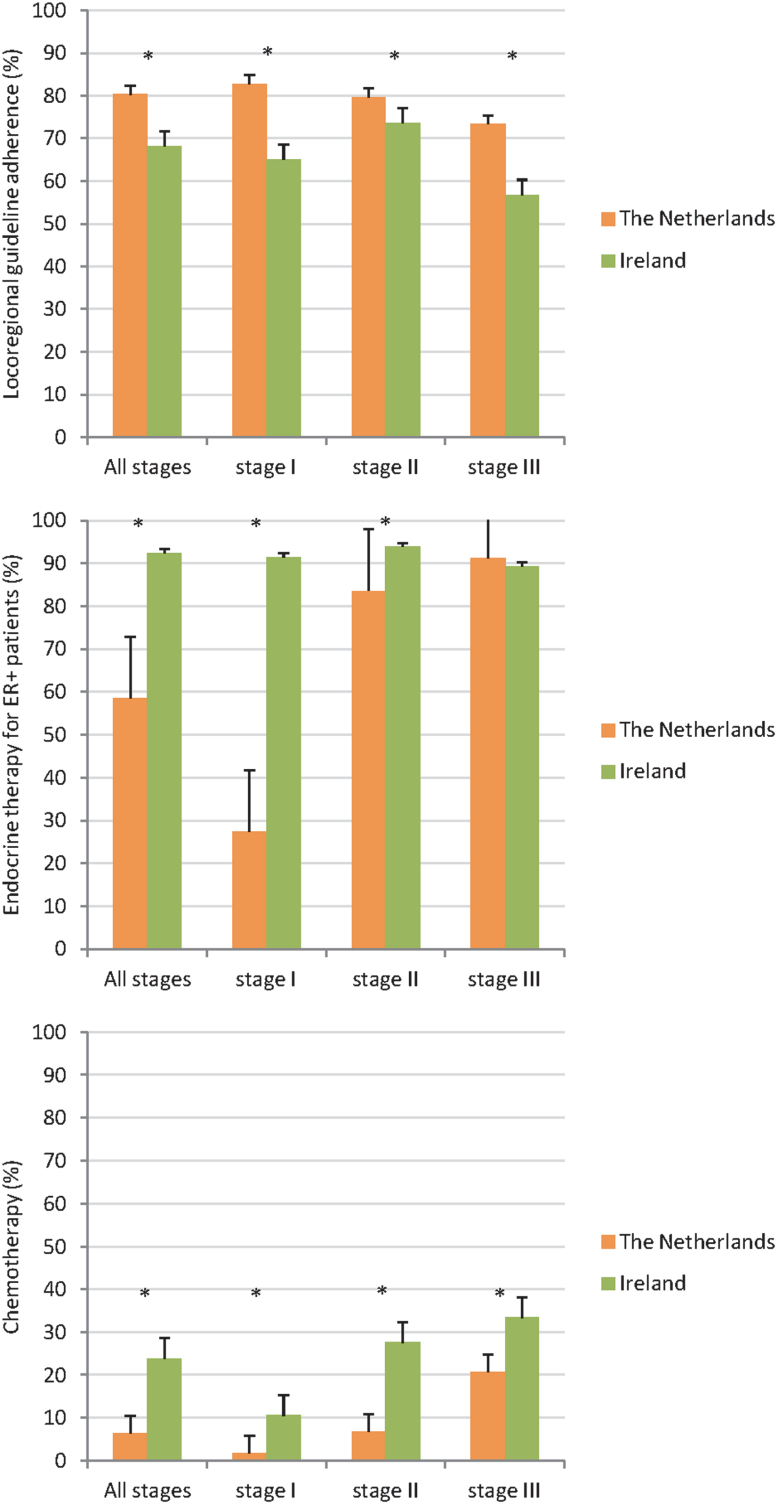
Treatment strategies. **A**. Locoregional guideline-adherence by stage. **B**. Endocrine therapy for estrogen receptor positive patients by stage. **C**. Chemotherapy by stage.

Fig. 1A shows the proportions of patients receiving guideline-adherent locoregional treatment by country, grouped by stage. In The Netherlands guideline-adherent treatment was performed in 80%, with little variation between stages, whereas these proportions in Ireland ranged from 57% (stage III) to 74% (stage II). Among patients who did not receive guideline-adherent locoregional treatment, 65% (The Netherlands) and 68% (Ireland), had no locoregional treatment at all, 6% (The Netherlands) and 13% (Ireland) had only BCS (without RT or axillary surgery), and 29% (The Netherlands) and 20% (Ireland) had adequate local treatment, but no axillary surgery. Adjusted RRs for having guideline-adherent locoregional therapy in Ireland relative to The Netherlands were 0.79 (95% CI 0.76–0.81), 0.87 (0.85–0.89) and 0.72 (0.68–0.75) respectively for stage I, II and III (*P*<0.001 for all stages).

Looking more specifically at locoregional treatment (Table 2), overall, more patients in Ireland had no breast surgery at all (19% vs. 12% in The Netherlands), also stratified by stage (*P*<0.001 in all stages).

**Table 2:** Treatment by stage.

	Country				
	The Netherlands		Ireland		
	N	%	N	%	P
All stages					
Definitive surgery					
None	4,971	12.1	1,121	19.2	<0.001
BCS	16,079	39.2	2,185	37.5	
Mastectomy	20,008	48.7	2,520	43.3	
Any axillary surgery	33,637	81.9	4323	74.2	<0.001
Radiotherapy					
All	19,407	47.3	2,940	50.5	<0.001
After BCS	15,050	93.6	1,728	79.1	<0.001
After Mastectomy	4,102	20.5	1,092	43.3	<0.001
Chemotherapy	2,638	6.4	138	23.8	<0.001
Endocrine therapy for ER+					
not imputed	11,570	58.5	3,609	88.6	<0.001
imputed (IRL)	11,570	58.5	3,764	92.4	<0.001
Stage I					
Definitive surgery					
None	1,341	7.5	258	15.6	<0.001
BCS	10,244	57.6	928	56.0	
Mastectomy	6,205	34.9	472	28.5	
Any axillary surgery	15,090	84.8	1,212	73.1	<0.001
Radiotherapy					
All	9,928	55.8	832	50.2	<0.001
After BCS	9,693	94.6	733	79.0	<0.001
After Mastectomy	209	3.4	86	18.2	<0.001
Chemotherapy	321	1.8	176	10.6	<0.001
Endocrine therapy for ER+					
not imputed	2,507	27.4	1,066	87.7	<0.001
imputed (IRL)	2,507	27.4	1,111	91.4	<0.001
Stage II					
Definitive surgery					
None	2,438	13.5	489	15.6	<0.001
BCS	5,226	29.0	1,135	36.1	
Mastectomy	10,359	57.5	1,516	48.3	
Any axillary surgery	14,665	81.4	2,486	79.2	0.004
Radiotherapy					
All	6,287	34.9	1,572	50.1	<0.001
After BCS	4,803	91.9	905	79.7	<0.001
After Mastectomy	1,443	13.9	632	41.7	<0.001
Chemotherapy	1,233	6.8	868	27.6	<0.001
Endocrine therapy for ER+					
not imputed	6,890	83.6	2000	89.6	<0.001
imputed (IRL)	6,890	83.6	2094	93.8	<0.001
Stage III					
Definitive surgery					
None	1,192	22.7	374	36.4	<0.001
BCS	606	11.6	122	11.9	
Mastectomy	3,444	65.7	532	51.8	
Any axillary surgery	3,882	74.1	626	60.9	<0.001
Radiotherapy					
All	3,192	60.9	536	52.1	<0.001
After BCS	554	91.4	90	73.8	<0.001
After Mastectomy	2,450	71.1	374	70.3	0.682
Chemotherapy	1,084	20.7	343	33.4	<0.001
Endocrine therapy for ER+					
not imputed	2,173	91.1	543	86.7	0.002
imputed (IRL)	2,173	91.1	559	89.3	0.188

In The Netherlands, 82% underwent any axillary surgical procedure, as compared to 74% in Ireland. Also, in all three stage groups, fewer patients in The Netherlands than in Ireland did not undergo axillary surgery (*P*<0.001).

Regarding radiotherapy (RT), among all patients, more patients received RT in Ireland than in The Netherlands, overall and after mastectomy (P<0.001). For mastectomy patients, the difference was only seen in stage I (18% of patients had post-mastectomy RT in Ireland vs. 3% in The Netherlands) and stage II (42% vs. 14%) (P<0.001). In stage III patients, the difference in the receipt of RT attenuated and no difference was observed in post-mastectomy RT. However, in all stages significantly fewer patients in Ireland received RT after BCS (79% vs. 94% in The Netherlands, overall, P<0.001). (Table 2).

### Endocrine therapy

The overall proportion of estrogen receptor positive patients receiving endocrine therapy differed between the countries—59% in The Netherlands vs. 92% in Ireland (*P*<0.001) for all stages combined. Patients with stage I disease were more than three times as likely to get endocrine therapy in Ireland (91% vs. 27%; *P*<0.001). The difference was smaller in stage II patients, 94% in Ireland vs. 84% in The Netherlands (*P*<0.001), and 89% vs. 91% respectively in stage III patients (P = 0.188) (Fig. 1B; Table 2). Adjusted RRs for having endocrine therapy in Ireland were 2.91 (95% CI 2.77–3.05), 1.11 (1.09–1.12) and 0.99 (0.96–1.02) respectively for stage I, II and III ER-positive patients. Among patients who did not receive any locoregional treatment at all, the proportions of endocrine monotherapy were 85% in The Netherlands and 86% in Ireland.

### Chemotherapy

Overall, 6% of patients The Netherlands and 24% of patients in Ireland received chemotherapy, and there was a higher proportion of Irish patients that received chemotherapy in all three stages (*P*<0.001) (Fig. 1C; Table 2). Adjusted RRs for having chemotherapy in Ireland were 4.55 (95% CI 3.81–5.43), 3.35 (3.11–3.62) and 1.44 (1.31–1.58), respectively for patients with stage I, II and III.

### Relative survival

Median follow-up time was 4.5 years for The Netherlands and 4.3 years for Ireland. During the total follow-up period, 14,771 (36.0%) patients died in The Netherlands, compared to 2,191 patients (37.6%) in Ireland.

**Fig 2:**
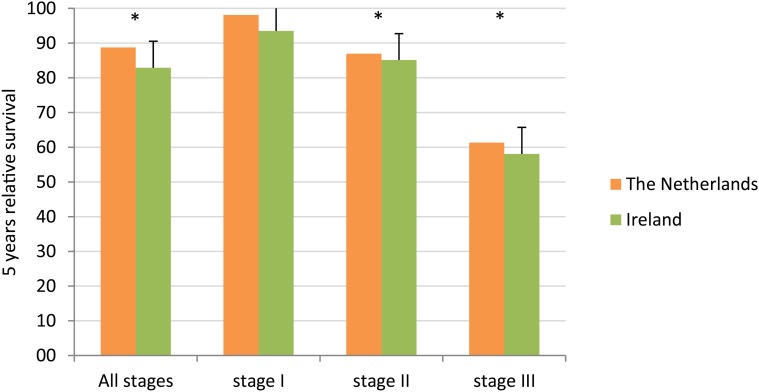
Relative survival by stage.

Five-year relative survival was 88.8% in The Netherlands and 82.9% in Ireland, for all stages combined (Fig. 2). This survival difference was statistically significant, also after adjustment for age, grade, stage, ER, PR and morphology (relative excess risk [RER] for Ireland, with The Netherlands as reference category: 1.22; 95% confidence interval (CI) 1.10–1.36). Grouped by stage, no survival difference was demonstrated in stage I patients (adjusted RER 1.00, 95% CI 0.59–1.70), but worse survival was confirmed for Irish patients in stage II (adjusted RER 1.20, 95% CI 1.02–1.42) and stage III (1.20, 95% CI 1.04–1.39).

## Discussion

The current study, comparing treatment and relative survival of older breast cancer patients between two Western European countries with similar treatment guidelines, showed large differences in treatment approach for older breast cancer patients. A higher proportion of patients in The Netherlands received guideline-adherent locoregional treatment than in Ireland in all stages, but in Ireland the receipt of systemic treatments was higher in all stages of disease. Relative survival of patients in Ireland was significantly poorer than in The Netherlands, but adjusted models suggested the difference most marked for stage II and III patients.

The observed discrepancies in breast cancer treatment are consistent with findings of earlier international comparisons of older and other breast cancer patients across Europe and the US [9,17–19]. However, although international survival and treatment variations among breast cancer patients have recently been assessed on a global scale [20], no clear evidence was published on the potential role of different treatment strategies in influencing survival discrepancies among early-stage cases. However, it is interesting to speculate on reasons for the differences in patterns of care for the specific treatment modalities. Some differences could be explained by discrepancies in guideline recommendations between the two countries. There are differences (S1 Appendix) especially for postmastectomy radiotherapy and chemotherapy and for both of these, indications are broader in the Irish guidelines. These differences are reflected in our results, where we observed more patients from Ireland receiving these treatments, as compared to the patients from Netherlands, also stratified by stage of disease. Secondly, physicians from the Netherlands may also be more likely to deviate from the guidelines when treating older breast cancer patients. Unfortunately, in our study it was also not possible to draw any reliable conclusion about the impact of differences in any of the specific treatment modalities, because of a potential bias due to confounding by indication when comparing the outcomes of patients with different treatments directly.

In a large population-based study in The Netherlands, guideline adherence of breast cancer treatment among younger and older breast cancer patients was compared between different regions, and although differences in adherence were observed, there were no significant survival differences between regions [21]. In the current study we found less guideline-adherence on locoregional treatment in Ireland, and this was accompanied by a worse survival in Ireland. On the other hand, patients in Ireland received more systemic therapies (both endocrine therapy and chemotherapy), so no conclusion can be drawn based on the locoregional treatment only, because of a probably counterbalanced effect by adjuvant treatments.

To obtain the highest level of evidence on treatment benefits, the effect of each treatment modality should be investigated based on randomized assignment of treatment. However, randomized clinical trials (RCTs) tend to be slow, expensive, and insensitive to the heterogeneous contexts of the general population [22]. The disadvantages of RCTs are probably even stronger in the older population, because of their limited mobility and large heterogeneity. Observational studies, using population-based registry data, are considered to be a better reflection of the “real world” [5,23]. However, although large study populations can be derived from registries, the observational design means that confounding by indication must be considered when studying treatment effects.

A limitation of our study was that the selected populations differed in some respects. Advanced stage and higher grade cases were more frequently observed in Ireland. Although the analyses included patients aged 65 and older, this finding might be explained partly by differences in screening [24,25] and possibly methods of grading between countries. To overcome the difference in stage distribution, we grouped all analyses by stage. Slight under-ascertainment of radiotherapy treatments is known to have occurred among Irish patients who had breast surgery in private hospitals. However, only about 17% of surgical patients in the age 65+ group falls into this category, and we estimate that the percentages of Irish patients reported as having radiotherapy in Ireland may about 2% too low, not enough to affect our conclusions.

To achieve best practice for older breast cancer patients, possibly, attention should be shifted to other outcomes rather than survival to improve quality of care for older breast cancer patients. However, we could draw no conclusions on aspects such as quality of life, risk of recurrence or complications, as we did not have data on these aspects. In addition, because of full anonymization of the datasets used for our analysis, characteristics of hospitals, such as the type (academic/teaching hospital, private/public clinic), but also the presence of radiotherapy facilities were not available. Therefore, we were unfortunately not able to see if guideline-adherence was associated with hospital characteristics.

The retrospective design of the current study, despite the positive arguments mentioned previously, remains a limitation. However, because of the availability of comprehensive cancer registry data, it was possible to create a large database of population-based, generalizable data.

In the future, study designs in which countries are compared on treatment strategy and breast cancer outcome are likely to be applied more frequently. By including many countries in analyses, specific populations that differ on only one treatment modality could be identified. Consequently, more evidence can be obtained from observational studies, by comparing patient outcomes between countries using an instrumental variable study design [26].

The European Registration of Cancer Care, or in short European Cancer Audit (EURECCA) aims [27] to create a population-based audit structure that covers all breast cancer patients across Europe: anonymous patient and tumor data, including treatment and outcome information will be registered in a uniform way across countries. The aim is to develop an extensive data source with the ultimate goal to define high-quality care and monitor the quality of care of all European cancer patients and so improving outcome of cancer care. EURECCA aims to investigate best practices and learn from them, as well as perform analysis on patient groups that deviate from guidelines such as the young and elderly. The availability of comprehensive cancer registry data, (like that used in the current study) facilitates the identification of large cohorts of population-based, generalizable data.

In conclusion, in this population-based study comparing patterns of care and survival of older breast cancer patients on a national scale in The Netherlands and Ireland, we found large differences in treatment approach, with more guideline-adherence on locoregional treatment in The Netherlands, and more prescription of systemic therapy in Ireland. Patients in Ireland had a worse relative survival as compared with the Dutch patients, although it was not possible to link this survival difference directly to differences in one or more of the specific treatment modalities. However, our finding should be a strong recommendation to perform more research on an international scale, with the ultimate goal to equalize the survival rates for breast cancer patients across Europe.

## Supporting Information

S1 Appendix Summary of guidelines. (DOCX)
