# Supplementary material for: Treatment Strategies and Survival of Older Breast Cancer Patients – An International Comparison between the Netherlands and Ireland
Source: PLoS One. 2015 Feb 3;10(2):e0118074. doi: 10.1371/journal.pone.0118074 (PMC4315587; doi:10.1371/journal.pone.0118074)
Supplement: S1 Appendix — (DOCX) [file pone.0118074.s001.docx]

| **Appendix S1 – Summary of guidelines** | | |
| --- | --- | --- |
|  | **The Netherlands** | **Ireland** |
| **Local surgery** | BCS: in all patients with T1-2; N0-1 breast cancer | BCS: unifocal, anticipated acceptable cosmetic result, not occurring in 1st/2nd trimester of pregnancy, possibility to obtain histologically clear margins (5mm), patient preference, no contraindications to RT |
|  | Mastectomy: patient preference  contraindications to BCS and/or RT | contraindications to BCS and/or RT |
| **Axillary surgery** | Sentinel node in all patients, except when axillary metastasis is proven, or with a ≥ T2 and/or multicentric primary tumor, or the patient had previous axillary surgery | 2000: SN still investigational, ALND (level I and II or I-III) in all patients  2007:  - sonographically normal LN's and cytology not performed/negative: SN  - proved LN involvement (by cytology or SN): axillary clearance (levels I and II or I-III) |
|  | Axillary lymph node dissection after positive SN or in case of contraindications to SN | 2000: SN still investigational, ALND (level I and II or I-III) in all patients  2007:  - sonographically normal LN's and cytology not performed/negative: SN  - proved LN involvement (by cytology or SN): axillary clearance (levels I and II or I-III)  After BCS |
| **Radiation therapy** | Always after BCS |  |
|  | After mastectomy: irradical resection, cT4, pT3 and ≥ pN2, involvement of pectoral muscle, or a positive axillary apex | After mastectomy in cases of high risk of local chest wall recurrence (tumor ≤3 mm of pectoral fascia or >4 positive axillary nodes) |
| **Endocrine therapy** | Postmenopausal N+ ER/PR+ | N+, ER/PR+ |
|  | N0, tumour size ≤ 1 cm: none | N0, tumour size ≤ 1 cm, ER/PR+, grade 1: none or tamoxifen |
|  | N0 tumour size 1-2 cm, grade 3  N0 tumour size 2-3 cm, grade 2-3  N0 tumour size ≥ 3 cm. | N0, tumour size ≥ 1 cm, ER/PR+, grade > 1 |
| **Chemotherapy** | Premenopausal N+ | All N0 ER/PR+ patients except "elderly": tumour size >1cm, all grades (before endocrine therapy) |
|  | Postmenopausal, age<70 N+ ER/PR-; | All N+ ER/PR- patients |
|  | Postmenopausal, age<60 N+ ER/PR+ in addition to endocrine therapy | Elderly: N+ and ER/PR- or tumour size>2 cm/grade 2/3 and any N-status |
|  | Postmenopausal, age<70 N+ ER/PR+, in addition to endocrine therapy when patient is motivated and fit, and prognosis is bad (i.e. ≥4 positive nodes) |  |
